# CdGAP maintains podocyte function and modulates focal adhesions in a Src kinase-dependent manner

**DOI:** 10.1038/s41598-022-21634-1

**Published:** 2022-11-04

**Authors:** Jun Matsuda, Dina Greenberg, Sajida Ibrahim, Mirela Maier, Lamine Aoudjit, Jennifer Chapelle, Cindy Baldwin, Yi He, Nathalie Lamarche-Vane, Tomoko Takano

**Affiliations:** 1 Division of Nephrology, McGill University Health Centre, 1001 Décarie, Montreal, QC Canada; 2 Cancer Research Program, Research Institute of the McGill University Health Centre, Montreal, QC Canada; 3 Department of Anatomy and Cell Biology, McGill University, Montreal, QC Canada

**Keywords:** RHO signalling, Podocytes

## Abstract

Rho GTPases are regulators of the actin cytoskeleton and their activity is modulated by GTPase-activating proteins (GAPs) and guanine nucleotide exchanging factors (GEFs). Glomerular podocytes have numerous actin-based projections called foot processes and their alteration is characteristic of proteinuric kidney diseases. We reported previously that Rac1 hyperactivation in podocytes causes proteinuria and glomerulosclerosis in mice. However, which GAP and GEF modulate Rac1 activity in podocytes remains unknown. Here, using a proximity-based ligation assay, we identified CdGAP (ARHGAP31) and β-PIX (ARHGEF7) as the major regulatory proteins interacting with Rac1 in human podocytes. CdGAP interacted with β-PIX through its basic region, and upon EGF stimulation, they both translocated to the plasma membrane in podocytes. CdGAP-depleted podocytes had altered cell motility and increased basal Rac1 and Cdc42 activities. When stimulated with EGF, CdGAP-depleted podocytes showed impaired β-PIX membrane-translocation and tyrosine phosphorylation, and reduced activities of Src kinase, focal adhesion kinase, and paxillin. Systemic and podocyte-specific CdGAP-knockout mice developed mild but significant proteinuria, which was exacerbated by Adriamycin. Collectively, these findings show that CdGAP contributes to maintain podocyte function and protect them from injury.

## Introduction

The Rho family of small GTPases (Rho GTPases) are master regulators of the actin cytoskeleton organization. Rho GTPases are molecular switches that shuttle between active (GTP-bound) and inactive (GDP-bound) forms. They are regulated by three families of Rho regulatory proteins: activating proteins called guanine nucleotide exchange factors (GEF) that exchange GDP for GTP^1^, inactivating proteins called GTPase-activating proteins (GAP) that enhance intrinsic GTPase activity^2^, and GDP dissociation inhibitors (GDI) that bind to and stabilize the inactive GDP-bound Rho GTPase forms in the cytoplasm^3,4^. Deciphering the molecular mechanisms regulating Rho GTPase activity is crucial for our understanding of cellular morpho-dynamics in many disorders, including kidney glomerular diseases.

The kidney glomerulus functions as a filtration barrier that retains large proteins and cells in the blood while allowing water and small molecules to pass into the urine. Proteinuria is a leakage of serum proteins into the urine and occurs when the glomerular filtration barrier is impaired. Proteinuria is one of the hallmarks of chronic kidney disease (CKD) and the degree of proteinuria often correlates with the rate of decline of kidney function. Thus, proteinuria is an important prognostic factor in kidney diseases.

Podocytes are highly specialized epithelial cells in the glomerulus, where they act as a critical component of the filtration barrier; injury to podocytes underlies many proteinuric glomerular diseases^5^. Podocytes have an intricate structure characterized by primary, secondary and tertiary projections. Finger-like tertiary projections, commonly known as “foot processes”, from adjacent podocytes interdigitate with one other and tightly surround glomerular capillary walls. The unique morphology of foot processes is supported by their well-organized actin cytoskeleton, and is critical for podocyte function^6^.

Indeed, Rho GTPases play a central role in podocyte morphology and attachment to the glomerular basement membrane (GBM). Of the 20 members, RhoA, Rac1, and Cdc42 are known as prototypical Rho GTPases and are the best studied^7^. The importance of the Rho GTPases has been well demonstrated by previous studies using podocyte-specific transgenic mice. For example, hyperactivation of Rac1 in podocytes causes proteinuria^8,9^, while loss of Rac1 can be situationally pathogenic or adaptive^10^. Meanwhile, gene deletion of Cdc42 in podocytes results in congenital nephrotic syndrome^10,11^.

To date, 82 GEFs, 69 GAPs and 3 GDIs have been identified in humans^1,2,12–14^, which act in concert to achieve cell-type and context-dependent dynamic balance of Rho GTPases. However, which and how Rho regulatory proteins regulate Rho GTPase activities in podocytes remains largely unknown.

In this study, we sought to investigate which Rho regulatory proteins interact with Rac1 in podocytes using proximity ligation combined with proteomics (BioID)^15^, and investigated their role in podocyte function and morphology. We reported recently that ARHGEF7 (β-PIX) is a predominant GEF that activates Cdc42^16^ in podocytes, and here we found that it is also an important Rac1 interactor. Furthermore, we identified Cdc42 GTPase-activating protein (CdGAP, also named ARHGAP31) as one of the major RhoGAPs that interacts with Rac1, but also with β-PIX, in podocytes. Given previous examples of CdGAP and GEF cooperation^17,18^, we explored the functional interaction of CdGAP and β-PIX in modulation of cell migration and adhesion of podocytes, and found that CdGAP facilitates β-PIX localization and activation at the cell membrane in a Src kinase-dependent manner upon EGF stimulation. Furthermore, we showed that CdGAP is required for maintenance of podocyte morphology and normal glomerular barrier function. These findings are pertinent to the identification of targets for small molecule therapeutics of kidney disease.

## Results

### CdGAP and β-PIX are the predominant regulators of Rac1 in podocytes

**Figure 1:**
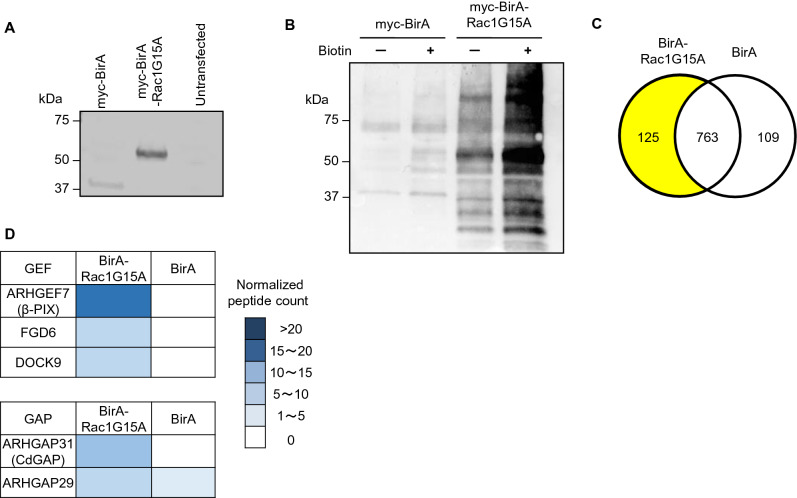
CdGAP and β-PIX interact with Rac1 in podocytes. (**A**) Doxycycline-inducible myc-BirA (control, 37 kDa) or myc-BirA-Rac1G15A (60 kDa) were stably expressed in cultured human podocytes. Both cell lines were treated with doxycycline for 16 h as described in Experimental Procedures. Representative immunoblot for myc is shown. (**B**) After 16 h incubation with biotin and doxycycline, presence of biotinylated proteins was verified by immunoblotting for streptavidin. (**C**) Venn diagram of proximity interactions identified in BirA-Rac1G15A expressing podocytes and controls expressing BirA alone. (**D**) Heatmap representing normalized peptide counts toward each bait of three GEFs and two GAPs, which are significantly enriched in BirA-Rac1G15A expressing podocytes compared to controls.

Rho GTPases interact dynamically with their regulators and effectors. To identify activators (GEFs) and inhibitors (GAPs) of Rac1 in podocytes, we performed proximity-based ligation and proteomics, BioID, using the G15A mutant of Rac1 (Rac1G15A) as bait^15,19^. Rac1G15A is a nucleotide-free mutant of Rac1 that binds to its GEFs with high affinity and, to a lesser extent, to its GAPs^20^. Rac1G15A was fused with a promiscuous *E.* *coli* biotin ligase (BirA) and expressed in immortalized human podocytes. Podocytes expressing BirA alone were used as controls (Fig. 1A). In the presence of exogenous biotin, proteins in close proximity to Rac1G15A were biotinylated, captured by streptavidin beads and subjected to mass spectrometry analyses (Fig. 1B)^15^. One hundred and twenty-five proteins were identified in BirA-Rac1G15A expressing podocytes at significantly higher levels compared to control podocytes expressing BirA alone (Fig. 1C). Cross-referencing these interactors to the list of known Rho regulatory proteins^2,12^, we identified three GEFs (ARHGEF7, FGD6, and DOCK9) and two GAPs (ARHGAP31 and ARHGAP29) (Fig. 1D). Adhesion of foot processes to the GBM via the adhesion molecules such as α3β1-integrins is critical for morphology and function of podocytes^21^. Therefore, of the two GAPs, CdGAP was of particular interest because of its known functions in the regulation of cell migration, adhesion and focal adhesions (FAs)^22–25^. Similarly, among the three GEFs, β-PIX is known to modulate FA maturation via Rac1 or Cdc42^26^, and to maintain podocyte architecture and glomerular function^16^.

### Loss of CdGAP enhances Rac1 and Cdc42 activities and impairs human podocyte cell morphology and motility

**Figure 2:**
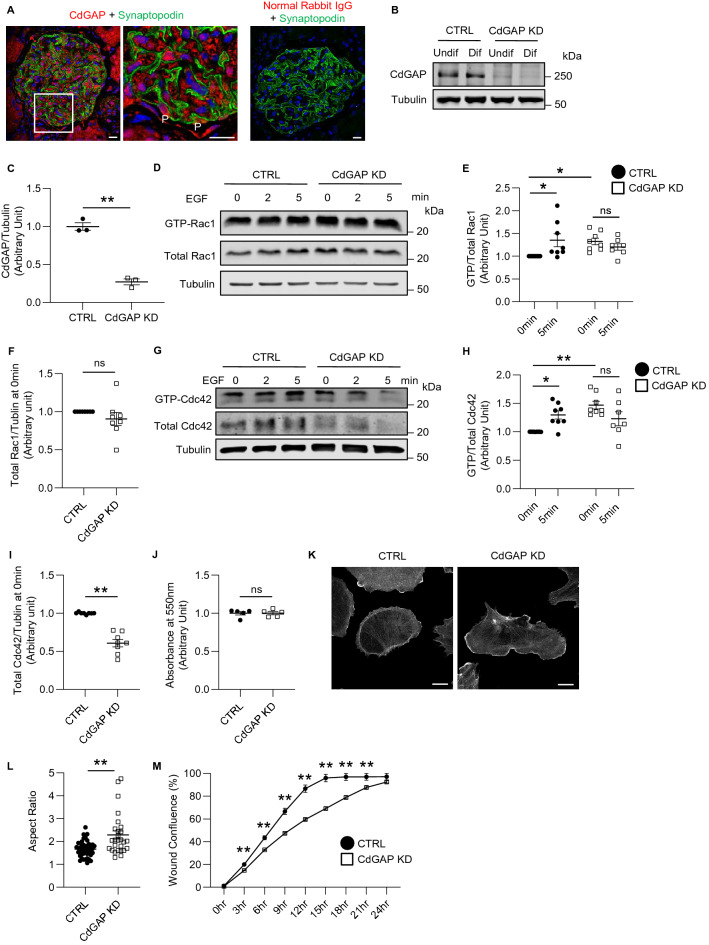
CdGAP modulates Rac1 activity, cell morphology, and motility in cultured podocytes. (**A**) Representative image of a glomerulus from a transplant donor kidney. Immunofluorescence staining was done for CdGAP (red) alongside the podocyte marker Synaptopodin (green) and DAPI (blue). The center panel shows a magnification of the indicated area (white squares) in the left panel. Normal rabbit IgG was used as a negative control (right panel). (**B**) Representative immunoblots for CdGAP and tubulin of cultured undifferentiated and differentiated human podocytes with CdGAP knockdown (KD) and controls (CTRL). (**C**) Densitometric quantification of CdGAP protein levels in differentiated podocytes in (**B**). (**D**,**G**) Cell lysates from differentiated CdGAP KD and control (CTRL) podocytes, which were untreated or treated with 100 ng/ml EGF, were subjected to pull-down with GST-CRIB for active GTP-bound forms of Rac1 (**D**) and Cdc42 (**G**)**.** Representative immunoblots for Rac1 (**D**) and Cdc42 (**G**) with tubulin are shown. (**E**,**H**) Densitometric quantification of active GTP-bound forms of Rac1 (**E**) and Cdc42 (**H**) normalized to total Rac1 and Cdc42, respectively. (**F**,**I**) Densitometric quantification of total Rac1 (**F**) and Cdc42 (**I**) protein normalized to tubulin. (**J**) Quantification of the attachment assay at 2 h after plating. The ratio of absorbance at 550 nm is shown. (**K**) Representative images of phalloidin staining of differentiated control (CTRL) and CdGAP knockdown (KD) podocytes. (**L**) Quantification of the aspect ratio in (**K**). (**M**) Quantification of undifferentiated podocyte migration in a scratch wound healing assay. n = 3 (**C**); 7 to 9 (**E** and **H**); 8 (**F** and **I**); 5 (**J**); 27 or 42 (**L**); 24 (**M**) in each group. ns, not significant. Statistically significant differences (*P < 0.05, **P < 0.01), assessed by either the Student’s t-test (**C**,**F**,**I**,**J**,**L,M**) or ANOVA with the Tukey–Kramer test (**E,H**) are indicated. Bar: 20 μm (**A,K**).

CdGAP is expressed widely in human and mouse tissues including the kidney^22,27,28^. To assess its expression in the glomerulus, we first performed immunofluorescence staining of a transplant donor kidney biopsy and confirmed CdGAP staining in podocytes, as well as in other glomerular cells and tubular epithelial cells (Fig. 2A). To study the role of CdGAP in human podocytes in vitro, we next established immortalized cultured human podocytes with CdGAP knockdown (KD) and their controls using short hairpin RNA (shRNA) lentiviruses. Immunoblotting confirmed a significant KD efficiency of 73% (Fig. 2B,C). We first assessed the effect of CdGAP depletion on the levels of Rac1 activity in differentiated podocytes by performing a GST-CRIB pull down assay. Loss of CdGAP resulted in a significant 1.33-fold increase in basal Rac1-GTP levels compared to control cells, without affecting the total levels of Rac1 (Fig. 2D–F). In control podocytes, Rac1 was activated after 5 min of EGF stimulation. However, further Rac1 activation by EGF was absent in CdGAP KD podocytes (Fig. 2D,E). Similarly, basal Cdc42 activity in CdGAP KD podocytes was significantly higher (1.47-fold) than in control cells, but CdGAP KD cells showed no EGF-induced Cdc42 activation (Fig. 2G,H). Curiously, total Cdc42 (normalized to tubulin) was significantly decreased in CdGAP KD podocytes (0.61-fold) compared with controls (Fig. 2I). When podocytes were plated onto a laminin-coated surface, both CdGAP KD and control podocytes attached similarly at 2 h (Fig. 2J), however, their cell morphology differed. In contrast to the archetypical rounded podocyte morphology, CdGAP-depleted podocytes showed an elongated cell shape with an increased aspect ratio (the ratio of the longest to the shortest axis of the cell) and enriched F-actin lamellipodia at the cell periphery (Fig. 2K,L, Supplementary Fig. S1), as previously published^29^. Furthermore, CdGAP KD podocytes were significantly slower to migrate compared to control cells in a wound healing assay over a period of 24 h (Fig. 2M, Supplementary Fig. S1). These results show that CdGAP acts as a GAP for Rac1 and Cdc42 in regulating podocyte morphology and migration but does not affect short-term cell adhesion.

### CdGAP interacts with β-PIX through the basic-rich region

**Figure 3:**
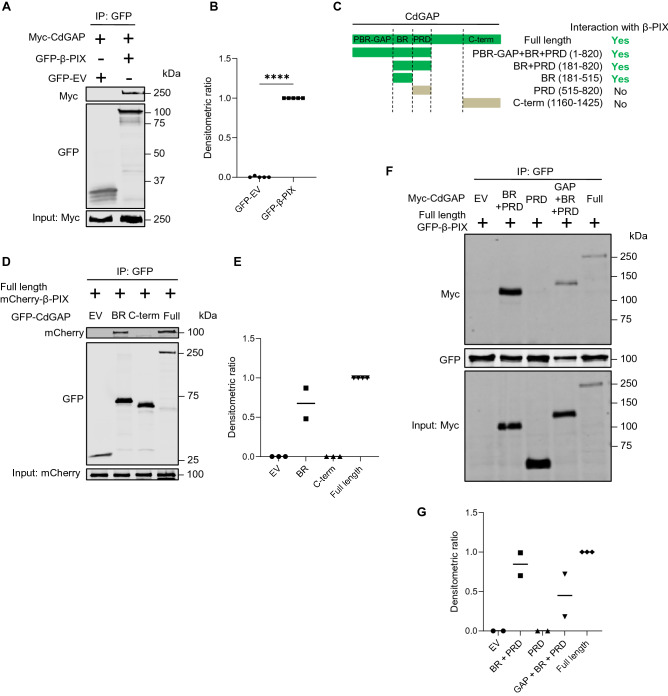
β-PIX interacts with the basic region of CdGAP. (**A**) HEK293 cells overexpressing myc-CdGAP and GFP-β-PIX were lysed and cell lysates were immunoprecipitated with anti-GFP antibody (for β-PIX) followed by immunoblotting for myc (for CdGAP). A representative immunoblot is shown. (**B**) Densitometric quantification of IP: normalized to total expression of myc-CdGAP in (**A**). (**C**) Schematic representation of the CdGAP deletion mutants, which were used in the co-immunoprecipitation experiments. (**D**) Cell lysates of HEK293 cells overexpressing GFP-CdGAP deletion proteins and full-length mCherry-β-PIX were immunoprecipitated with anti-GFP antibody (for CdGAP) followed by immunoblotting for mCherry (for β-PIX). (**E**) Densitometric quantification of IP: normalized to total mCherry-β-PIX expression in (**D**). (**F**) Cell lysates of HEK293 cells overexpressing myc-CdGAP deletion proteins and full-length GFP-β-PIX were immunoprecipitated with anti-GFP antibody (for β-PIX) followed by immunoblotting for myc (for CdGAP). (**G**) Densitometric quantification of IP: normalized to total myc-CdGAP expression in (**F**). n = 5 (**B**); 2–4 (**E**), 2–3 (**C**). Statistically significant differences (****P < 0.0001), assessed by the Student’s t-test test (**B**), are indicated. EV: Empty vector; BR: basic-rich; PRD: proline-rich domain; C-term: C-terminus.

We have previously reported that the Cdc42 GEF intersectin (ITSN) interacted with CdGAP and inhibited its GAP function^17,18^. By analogy, we questioned if β-PIX and CdGAP, both found in the proximity of Rac1G15A in podocytes by BioID (Fig. 1), interacted with each other. When myc-CdGAP and GFP-β-PIX were transiently transfected in HEK293 cells and β-PIX was immunoprecipitated using anti-GFP antibodies, CdGAP co-immunoprecipitated, indicating that CdGAP and β-PIX bound one another, either directly or indirectly (Fig. 3A,B). We found that co-immunoprecipitation of CdGAP and β-PIX was not altered by EGF treatment of HEK293 cells (Supplementary Fig. S2). Attempts to demonstrate interaction between endogenous CdGAP and β-PIX in podocytes were unsuccessful due to nonspecific interactions detected in the rabbit immunoglobulin G negative control (Supplementary Fig. S2).

CdGAP consists of a polybasic region (PBR) preceding the GAP domain, a basic central region (BR), a proline-rich domain (PRD), and an extended C-terminal regulatory domain (Fig. 3C)^17,30^. To determine which part of CdGAP is required for the interaction with β-PIX, we tested a series of CdGAP deletion constructs in co-immunoprecipitation assays with β-PIX (Fig. 3C). CdGAP constructs containing the BR domain [(BR alone (181–515), BR + PRD (181–820), and PBR-GAP + BR + PRD (1–820)] co-immunoprecipitated with the full-length β-PIX, while the PRD alone (515–820) and the C-terminus domain (1160–1425) failed to do so (Fig. 3D–G). Therefore, the BR domain of CdGAP is required and sufficient for the interaction with β-PIX.

### CdGAP modulates subcellular localization of β-PIX in podocytes

**Figure 4:**
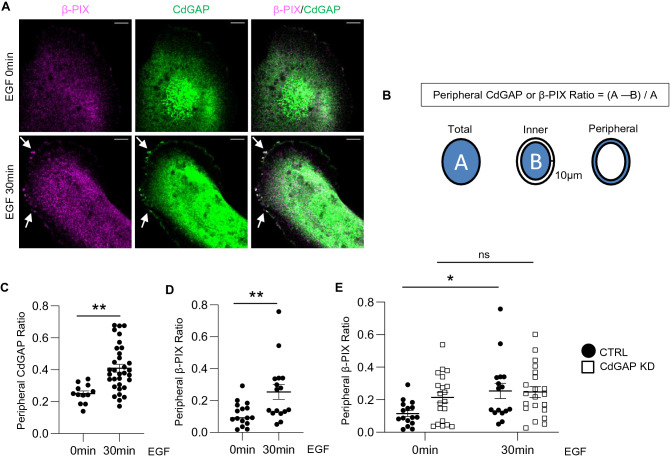
CdGAP and β-PIX interact and translocate to the podocyte periphery upon EGF stimulation. (**A**) Representative confocal microscopy images of the immunofluorescence staining for β-PIX (magenta) and CdGAP (green) using differentiated control (CTRL) podocytes treated with EGF (100 ng/ml) for 0 min and 30 min. Areas of colocalization at the cell periphery are demarcated by arrows. (**B**) Quantification method of the peripheral CdGAP or β-PIX ratio is outlined. (**C** and **D**) Quantified peripheral CdGAP (**C**) and β-PIX (**D**) ratio. (**E**) Comparison of peripheral β-PIX ratio between differentiated CdGAP KD and CTRL podocytes treated with EGF. CTRL values are the same as in (**D**) and included for comparison. n = 12 or 33 (**C**); 16 (**D**); 16 to 21 (**E**) in each group. ns, not significant. Statistically significant differences (*P < 0.05, **P < 0.01), assessed by either the Student’s t-test (**C,D**) or ANOVA with the Tukey–Kramer test (**E**), are indicated. Bar: 10 μm (**A**).

The above findings indicate that there is a protein–protein interaction, either direct or indirect, between β-PIX and CdGAP. CdGAP KD human podocytes have some phenotypic similarity (e.g. impaired motility; Fig. 2) to that of β-PIX KD mouse podocytes^16^. Thus, we hypothesized that CdGAP may modulate the function of β-PIX in podocytes. First, we studied whether CdGAP impacts subcellular localization of β-PIX by immunocytochemistry. In control podocytes, β-PIX and CdGAP were predominantly localized in the cytosol (Fig. 4A). When cells were stimulated with EGF for 30 min, translocation of both CdGAP and β-PIX to the plasma membrane was observed (Fig. 4A, quantified in Fig. 4B–D). When the overlap of CdGAP and β-PIX was quantified using the Pearson correlation coefficient, a modest but significantly increased overlap at the cell periphery (10 μm within the plasma membrane) was observed after EGF stimulation, while overlap in the whole cell was unchanged (Supplementary Fig. S3). Of interest, in CdGAP KD podocytes, the peripheral β-PIX ratio did not increase by EGF stimulation in contrast to control podocytes (Fig. 4E). Therefore, these results suggest that upon EGF stimulation, CdGAP translocates to the plasma membrane and facilitates membrane translocation of β-PIX.

### CdGAP modulates focal adhesion size and β-PIX /FAK/paxillin tyrosine phosphorylation in a Src kinase-dependent manner in podocytes

**Figure 5:**
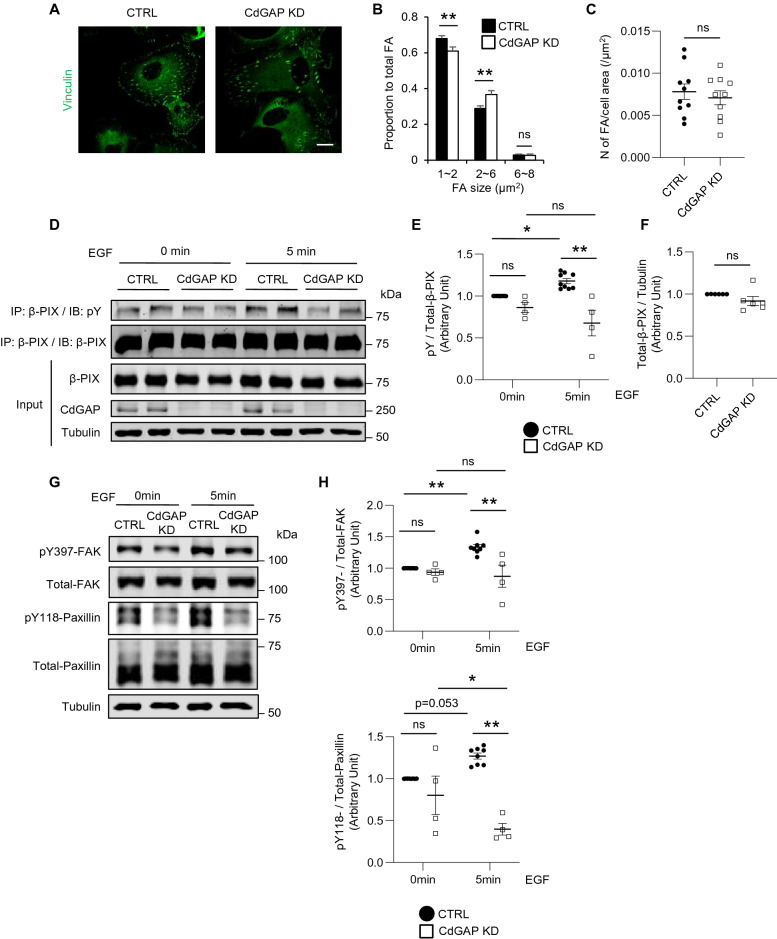
CdGAP modulates focal adhesions in podocytes. (**A**) Representative images of the immunofluorescence staining for vinculin (green) of differentiated CdGAP KD and control (CTRL) podocytes. (**B**) The proportion of small (< 2 µm^2^), medium (2–6 µm^2^), and large (< 6 µm^2^) focal adhesions (FA) to total FA is shown. (**C**) The number of FA per cell area. (**D**) Differentiated CdGAP KD and CTRL podocytes were stimulated with EGF (100 ng/ml) and lysed at the indicated times. Lysates were subjected to co-immunoprecipitation with anti-β-PIX antibody, followed by immunoblotting for phosphotyrosine (pY). Representative immunoblots for pY, β-PIX, CdGAP, and tubulin are shown. (**E**) Densitometric quantification of pY normalized to total expression of β-PIX in (**D**). (**F**) Densitometric quantification of total expression of β-PIX normalized to tubulin in (**D**). (**G**) Representative immunoblots for pY- and total FAK and paxillin using differentiated CdGAP KD and CTRL podocytes treated with EGF (100 ng/ml). (**H**) Densitometric quantification of pY397-FAK and pY118-paxillin normalized to total FAK and paxillin, respectively, in (**G**). n = 16–20 (**B**); 10 (**C**); 4–9 (**E**); 6 (**F**): 4–8 (**H**). ns, not significant. Statistically significant differences (*P < 0.05, **P < 0.01), assessed by either the Student’s t-test (**C,F**) or ANOVA with the Tukey–Kramer test (**E,H**), are indicated. Bar: 20 µm (**A**).

FA is an integrin-based multi-protein complex and is essential for podocyte attachment to the GBM and its motility^31^. Pro-migratory and pro-invasive functions were ascribed to CdGAP, which was shown to regulate directional membrane protrusions of migrating osteosarcoma cells^23,29^, breast cancer cells^24^, and prostate cancer cells^25^. Consistently, CdGAP KD podocytes showed decreased motility (Fig. 2M). Thus, we next studied FA size in CdGAP KD podocytes. When FAs were visualized by immunostaining of vinculin, FAs in CdGAP KD podocytes were generally larger in size than those of control cells (Fig. 5A,B), while the number of FAs per cell area was comparable (Fig. 5C). It has previously been shown that tyrosine phosphorylation of β-PIX by EGF leads to its activation and increases FA turnover^26,32^. To assess tyrosine phosphorylation of β-PIX, β-PIX was immunoprecipitated from control and CdGAP KD podocytes and immunoblotted for phospho-tyrosine (pY). Basal tyrosine phosphorylation of β-PIX was observed in unstimulated podocytes, and increased further by EGF stimulation (Fig. 5D,E). Depletion of CdGAP in podocytes did not alter basal tyrosine phosphorylation of β-PIX but rendered KD cells unresponsive to further stimulation (Fig. 5D,E). In fact, β-PIX tyrosine phosphorylation after EGF stimulation was significantly lower in CdGAP KD podocytes compared with control podocytes (Fig. 5E), while total amounts of β-PIX were unchanged between control and CdGAP KD podocytes (Fig. 5F). These results indicate that EGF-induced β-PIX tyrosine phosphorylation and activation is inhibited in the absence of CdGAP. Like β-PIX, many FA proteins become active when tyrosine-phosphorylated and this in turn results in FA turnover^33^. Thus, we studied the tyrosine phosphorylation of the FA molecules, focal adhesion kinase (FAK) and paxillin. In control podocytes, basal pY of FAK (pY397) and paxillin (pY118) was observed, which significantly increased upon EGF stimulation (Fig. 5G,H). In CdGAP KD podocytes, basal pY was unchanged but the response to EGF was absent (Fig. 5G,H). The lack of increase of pY397-FAK and pY118-paxillin in response to EGF in CdGAP KD cells, which correlated with the ablated tyrosine phosphorylation of β-PIX (Fig. 5E), was specific since phosphorylation/activation of ERK in response to EGF was intact in these cells (Supplementary Fig. S4). These results demonstrate that activation of FA proteins (such as β-PIX, FAK and paxillin), in response to ligand stimulation (e.g. by EGF), is impaired in CdGAP KD podocytes, and this likely results in the increase of larger FAs.

**Figure 6:**
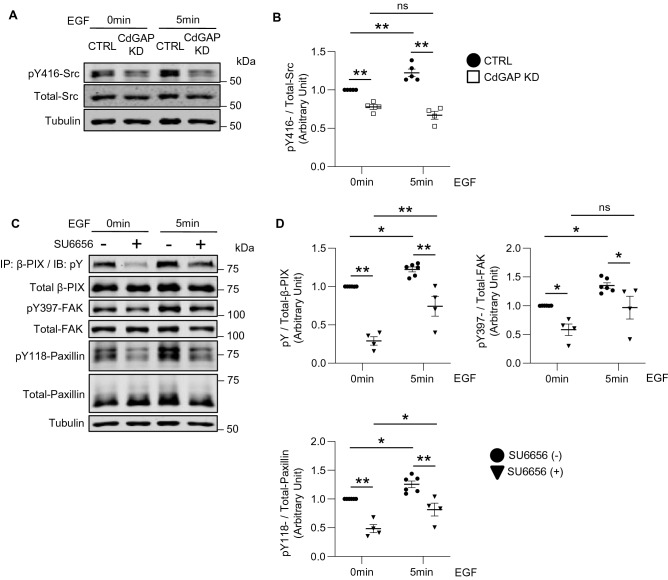
CdGAP is required for EGF-induced Src kinase activity in podocytes. (**A**) Representative immunoblots for pY416 and total Src using CdGAP KD and control (CTRL) podocytes treated with EGF (100 ng/ml). (**B**) Densitometric quantification of pY416 Src normalized to total Src in (**A**). (**C**) Representative immunoblots for pY- and total β-PIX, FAK and paxillin using CTRL podocytes. Cells were treated with vehicle or 5 µM Src inhibitor, SU6656 for 16 h before EGF stimulation. (**D**) Densitometric quantification of pY-β-PIX, pY397-FAK and pY118-paxillin normalized to total expression in (**C**). n = 4 or 5 (**B**); 4 or 6 (**D**) in each group. Statistically significant differences (*P < 0.05, **P < 0.01), assessed by ANOVA with the Tukey–Kramer test (**B,D**), are indicated.

The above findings suggest that the absence of CdGAP blocks a signaling pathway that leads from EGF/EGFR interaction to β-PIX/FAK/paxillin tyrosine phosphorylation downstream of or in parallel with ERK activation, most likely implicating protein tyrosine kinase(s). A strong candidate is the Src-family tyrosine kinase because of its well-known role in tyrosine-phosphorylation of β-PIX, FAK, and paxillin^32,33^. Src activity requires autophosphorylation of its Y416^34^. Basal pY416-Src, representing active Src, was significantly decreased in CdGAP KD cells compared to controls, and did not increase in response to EGF stimulation (Fig. 6A,B). Thus, both basal and EGF-stimulated Src activity are suppressed in the absence of CdGAP. We next treated podocytes with the Src inhibitor, SU6656. Basal tyrosine phosphorylation of β-PIX, FAK, and paxillin were significantly decreased in treated group, and while all three responded to EGF stimulation, EGF-stimulated pY of β-PIX, FAK, and paxillin was lower in SU6656 treated cells compared to controls (Fig. 6C,D), phenocopying CdGAP KD podocytes (Fig. 5D–H). Our findings suggest that CdGAP modulates FAs through (1) its GAP activity towards Rac1/Cdc42, and (2) facilitation of Src kinase activity, which contributes to the tyrosine phosphorylation and subsequent activation of FA proteins such as β-PIX, FAK, and paxillin.

### CdGAP in podocytes is required for glomerular barrier function in adult mice

**Figure 7:**
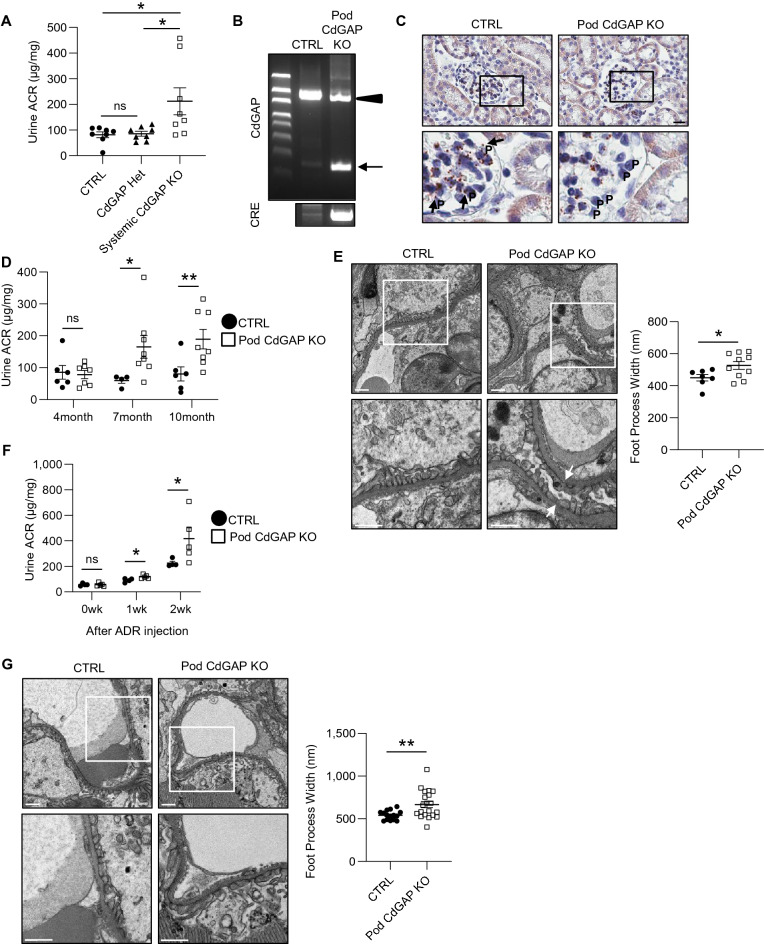
CdGAP in podocytes is required for glomerular barrier function in adult mice and has a protective role against injury. (**A**) Quantification of urine ACR from 6-month-old systemic CdGAP deficient (KO), heterozygous (Het), and control (CTRL) mice. (**B**) Representative PCR analysis of the extracted DNA obtained from isolated glomeruli of podocyte-specific CdGAP deficient (CdGAP^Pod-/-^; Pod KO) and CTRL mice. PCR product bands of podocyte-specific excision (321 bp; arrow) and non-excised loxP flanked sequence (1328 bp; arrowhead) derived from other glomerular cells in CdGAP^Pod-/-^ mice are shown. (**C**) Representative images of in situ hybridization for CdGAP mRNA in the kidney. Black arrows show CdGAP mRNA in podocytes of CTRL mice. (**D**) Quantification of urine ACR from 4-month-old, 7-month-old, and 10-month-old CdGAP^Pod-/-^ and CTRL mice. (**E**) Representative electron microscopy of the kidney from 12-month-old CdGAP^Pod-/-^ and CTRL mice (left) and quantification of foot process width (right). White arrows show focal effacement of foot processes in CdGAP^Pod-/-^ mice. (**F**) Quantification of urine ACR from CdGAP^Pod-/-^ and CTRL mice before and at 1 week and 2 weeks after ADR injection. (**G**) Representative electron microscopy of the kidney from CdGAP^Pod-/-^ and CTRL mice at 4 weeks after ADR injection (left) and quantification of foot process width (right). (**C**,**E,G**) The lower panels show a magnification of the indicated areas (black and white squares) in the top panels. n = 8 (**A**); 4 to 8 (**D**); 7 or 10 (**E**); 4 or 5 (**F**); 15 or 20 (**G**) in each group. ns, not significant. Statistically significant differences (*P < 0.05, **P < 0.01), assessed by either ANOVA with the Tukey–Kramer test (**A**) or the Student’s t-test (**D-G**), are indicated. Bars: 20 μm (**C**); 1 μm (**E,G**). P: Podocyte (**C**).

Since CdGAP is required to maintain podocyte function in vitro, we next interrogated the role of CdGAP in the glomerular barrier function using systemic CdGAP knockout (KO) mice. We reported previously that mice with systemic CdGAP KO have 44% embryonic/perinatal mortality resulting from vascular defects, but survivors grow normally and are fertile^27^. To assess the role of CdGAP in the glomerular barrier function, we collected urine from systemic CdGAP KO mice and littermate controls and quantified the albumin-to-creatinine ratio (ACR). Male systemic CdGAP KO mice developed mild but significant proteinuria starting at around 6 months old (Fig. 7A). Female mice did not develop proteinuria up to 12 months old (data not shown). The susceptibility of males to proteinuria has been well recognized both in humans and mice^35^ and we have observed a similar sex difference of proteinuria in podocyte specific Ste20-Like Kinase (SLK) transgenic mice^36^.

Proteinuria can occur from defects of any of the three components of the glomerular filtration barrier; namely glomerular endothelial cells, GBM, and podocytes. To determine whether loss of CdGAP in podocytes is responsible for proteinuria, we next generated podocyte-specific CdGAP knockout (Pod KO hereafter) mice by crossing Podocin-Cre mice with CdGAP^*flox/flox*^ mice. PCR of genomic DNA extracted from isolated glomeruli confirmed a truncated gene deletion product of CdGAP in Pod KO mice (Fig. 7B). Since we could not identify the antibody that reliably detects CdGAP expression in podocytes in the mouse kidney by immunostaining, we assessed the expression of CdGAP mRNA in the glomerulus using in situ hybridization. CdGAP mRNA was observed predominantly in the endothelial cells, consistent with previous reports^37^. CdGAP mRNA was also observed in the glomerular podocytes in control mice, but not in KO mice (Fig. 7C), consistent with the successful podocyte-specific deletion of CdGAP. Male, but not female Pod KO mice developed significant proteinuria at around 7 months of age, phenocopying systemic CdGAP knockout mice (Fig. 7D). While Pod KO mice did not show discernible abnormalities by kidney histology (PAS staining) and serum biochemistry (blood urea nitrogen [BUN] and creatinine) up to 12 months old (data not shown), electron microscopy demonstrated that foot process width was significantly increased in 12-month-old male Pod KO mice compared with controls, indicating foot process effacement (Fig. 7E).

To study whether young Pod KO mice are more susceptible to injury, we challenged 14–16-week-old male Pod KO and control mice with a podocyte toxin, Adriamycin (ADR). Urine ACR was increased after ADR injection in both groups, but Pod KO mice showed significantly higher ACR than control mice at 1 and 2 weeks (Fig. 7F). In addition, we found significantly more foot process effacement in Pod KO mice compared with controls at 4 weeks (Fig. 7G). Altogether, these findings indicate that CdGAP in podocytes is required for normal glomerular barrier function, and its absence makes podocytes susceptible to external injury.

## Discussion

Previous studies, including ours, have established that Rac1 plays pathogenic or adaptive/protective roles in podocytes depending on the context^8–10^. Thus, our study aimed to ascertain which GAPs and GEFs regulate Rac1 activity in podocytes. Using Rac1 as bait in BioID, we identified CdGAP as one of the GAPs that interacts with Rac1. CdGAP is one of the causative genes associated with Adams-Oliver syndrome, a disorder characterized by scalp defects and terminal transverse limb abnormalities^38^. CdGAP localizes at FAs and participates in the regulation of cell motility and adhesion dynamics^23^, and its roles in cancer cells have been well described^24,25,29,39^. While CdGAP was initially discovered as a GAP for Cdc42, it has been since shown that it acts on both Cdc42 and Rac1^20,22^. In the current study, CdGAP KD podocytes showed increased basal activities of Rac1 and Cdc42, indicating that CdGAP suppresses both Rac1 and Cdc42 activities under basal conditions. This is consistent with previous reports using U2OS cells with CdGAP KD or an inactive CdGAP mutant^23,29^.

GEFs and GAPs act in concert in a cell-/context-dependent manner. We reported recently that β-PIX is a predominant GEF for Cdc42 in podocytes and facilitates its downstream YAP activity^16^. The present study demonstrated that β-PIX interacts not only with Cdc42 but also with Rac1, and furthermore with CdGAP in podocytes. The existence of such GEF-GAP interactions is supported by a recent study showing that Rho regulatory proteins can form both homotypic and heterotypic interactions, suggesting that these complexes can potentiate the ability of GEFs and GAPs to coordinate downstream Rho GTPase activities^40^. One study identified a GAP-GEF interaction between SrGAP and β-PIX that facilitates crosstalk between Cdc42 and RhoA in a collagen-dependent manner^41^. Similarly, we previously showed that CdGAP interacts with another GEF, intersectin (ITSN), in fibroblasts via its BR domain^17,18^, which is the same domain that is required for binding to β-PIX in the current study. In the case of the ITSN-CdGAP interaction, the binding of ITSN inhibits the GAP activity of CdGAP, facilitating Cdc42 activation^17^. Here, we also observed a synergy between β-PIX and CdGAP. When CdGAP was depleted in podocytes, basal Rac1 and Cdc42 activities were increased compared to CdGAP intact podocytes, likely reflecting the diminished GAP activity of CdGAP toward Rac1 and Cdc42. However, the most striking phenotype was that CdGAP KD podocytes did not respond to EGF in the activation of Rac1 nor Cdc42. Similarly, EGF-induced membrane translocation and tyrosine phosphorylation of β-PIX were absent in CdGAP KD podocytes. Thus, CdGAP is required for the functional EGF-mediated translocation and activation of β-PIX. Our previous study showed that CdGAP translocated to the plasma membrane in response to platelet-derived growth factor and this was dependent on the N-terminal PBR domain^30^. It is possible that EGF also triggers membrane translocation of CdGAP in a similar manner and β-PIX in complex with CdGAP moves together, where it gets tyrosine phosphorylated. Whether CdGAP-β-PIX interaction is direct or indirect is yet to be determined.

β-PIX and other FA proteins such as FAK and paxillin are known to be tyrosine-phosphorylated by the Src-family kinases^32,42^. A previous study clearly demonstrated that Src, in turn, is activated by mechanical stimuli via integrin at the FA^43^. CdGAP is a key mediator of FA-based mechanosensing of extracellular matrix^23,29^, thus it appears reasonable to postulate that CdGAP plays an important role in activating Src family kinases especially in FAs. Indeed, we observed a low Src kinase activity in CdGAP KD cells, which was unresponsive to EGF stimulation. The consistent lack of response to EGF stimulation in CdGAP KD cells highlights the potential role of CdGAP in FA-based mechanosensing and consecutive signal transduction. Similar findings were observed in our previous study, which showed that CdGAP deletion in endothelial cells impaired VEGF-induced tyrosine phosphorylation of Gab1 and its downstream signaling pathways^27^. However, given that pY-β-PIX/FAK/paxillin in Src inhibitor-treated podocytes was blunted basally but remained partially responsive to EGF stimulation, it is likely that Src is not the sole regulator of the signaling pathway responsible for EGF-mediated tyrosine phosphorylation of β-PIX, FAK, and paxillin.

Collectively, our findings support a model wherein CdGAP interacts with β-PIX in the cytoplasm and contributes to maintaining low Rac1/Cdc42 activity at basal conditions. In response to external stimuli, CdGAP and β-PIX are recruited to the plasma membrane, where they collaborate to activate Rac1/Cdc42 and regulate FA proteins in a Src kinase dependent-manner. In the absence of CdGAP, excess activation of β-PIX/Rac1/Cdc42 under basal conditions, or impaired response to external stimuli in pathological conditions, may be detrimental to podocyte health.

Our findings in vivo support the importance of CdGAP in the maintenance of FAs in podocytes. We demonstrate that CdGAP deletion in glomerular podocytes exacerbated proteinuria in mice, both under basal conditions and within a podocyte injury model. The basolateral membrane of podocyte foot processes is anchored to the underlining GBM via a number of adhesion molecules represented by α3β1-integrins^44^. The importance of the proteins that interact with the cytoplasmic domain of integrins (collectively called adhesion complex proteins) has been reported in podocyte function^45–47^. Thus, it is likely that the absence of CdGAP in glomerular podocytes disrupts FA complexes, thereby impairing GBM-podocyte interaction and disrupting integrin-linked actin cytoskeleton regulation.

In conclusion, CdGAP is required for podocyte function and is protective from injury in vivo. Improving our understanding of the CdGAP-mediated Src kinase activation that likely takes place downstream of integrins and FA complexes in podocytes may allow for the development of a novel approach to proteinuric kidney diseases.

## Methods

### Cell culture and transient transfection

Immortalized human podocytes (gifts from Dr. Moin Saleem)^48^ were cultured in RPMI1640 containing 10% fetal bovine serum (FBS) and 1% penicillin/streptomycin (Gibco) at permissive conditions (33 °C in 5% CO_2_). Podocytes with CdGAP KD and their controls were established using MISSION Lentiviral shRNA (Sigma-Aldrich, TRCN0000047640 and SHC001, respectively). HEK293T cells were transiently transfected using the lentiviral packaging system (abm, LV003) according to the manufacturer’s instructions. Virus-containing supernatants were added to podocytes under permissive conditions for 16 h. Puromycin (Wisent Inc.) was added 48 h later and puromycin-resistant cells were pooled for further experiments. Unless stated otherwise, podocytes were differentiated under non-permissive condition (37 °C in 5% CO_2_) for 7 days, as described previously^48^.

### Antibodies and reagents

The antibodies and reagents used are summarized in Supplemental Table S1.

### BioID/proteomics

Myc-BirA-Rac1G15A (from Dr. Jean-Francois Cote, Montreal Clinical Research Institute) was subcloned into pTRE2 (Clontech) and expressed in immortalized human podocytes that stably express rtTA using nucleofection^48^. As control, pTRE2-myc-BirA was used. BioID experiment was performed as described previously^16^. Briefly, cells were incubated for 16 h with exogenous 50 μM biotin (BioShop, BIO302) and doxycycline (Sigma, D9891) and biotinylated proteins were captured by streptavidin beads (Thermo Scientific) and subjected to proteomics analyses. The results were analyzed by the Scaffold Q + Scaffold_4.9.0 software (Proteome Sciences). For statistics, t-test within the Scaffold was used after normalizing to total spectral counts. All proteins with a *P*-value of < 0.05 and a fold change of two over the BirA controls were considered as interactors of BirA-Rac1G15A. The mass spectrometry proteomics data have been deposited to the ProteomeXchange Consortium via the PRIDE (Perez-Riverol et al., 2019) partner repository with the dataset identifier PXD023718.

### Immunoblot analysis

Cultured podocytes and HEK293 cells were lysed with ice-cold lysis buffer (10 mM Tris [pH 7.5], 1 mM EDTA [pH 8.0], 1 mM EGTA [pH 8.0], 125 mM sodium chloride, 10 mM sodium pyrophosphate, 25 mM sodium fluoride, 2 mM sodium orthovanadate, 1% Triton X-100 [Sigma-Aldrich], and protease inhibitor cocktail [Roche]). Protein concentrations were measured and quantitative densitometry was performed as previously described^16^. Lysates were assayed by Western blot.

### Histological analysis

Histological analysis was performed as previously described^16^. Human kidney sections were obtained from a kidney transplantation donor. Mice were transcardially perfused with PBS. Tissues were post-fixed with 4% paraformaldehyde (PFA) (Thermo Fisher Scientific) and embedded in paraffin. Paraffin sections were immunostained with the respective antibodies. Citrate treatment was performed for antigen retrieval. Tissue sections were counterstained with 4,6-diamidino-2-phenylindole (DAPI) (Invitrogen). Immunofluorescence images were obtained using a Zeiss LSM780 laser scanning confocal microscope with the Zeiss Plan Apochromat 63 × 1.40 Oil DIC objective. All imaging parameters were maintained constant throughout image acquisition of all samples.

### Immunofluorescence

Undifferentiated podocytes were plated on glass cover slips coated with 0.25 μg/cm^2^ laminin 521 (CORNING) and allowed to differentiate for 7 days. They were then fixed in 4% PFA before being permeabilized with 0.1% Triton. After blocking with 3% bovine serum albumin in PBS, cells were immunostained with the respective antibodies and stained with phalloidin. Immunofluorescence images were obtained using a Zeiss LSM780 laser scanning confocal microscope with the Zeiss Plan Apochromat 63 × 1.40 Oil DIC objective. All imaging parameters were maintained constant throughout image acquisition of all samples. Quantification of FA complex was done using ImageJ software, as described previously^49^. Briefly, cell contour was traced using phalloidin-stained images with the ‘Freehand Line’ function and cell area was measured. After shifting to images with vinculin staining, the ‘Clear Outside’ function was used to erase the area outside the cell region. The number of particles between 1 and 8 μm^2^ was counted. FAs were classified into three groups: small (1–2 μm^2^), medium (2–6 μm^2^), and large (6–8 μm^2^). The proportion of the number of FAs in each group relative to total FAs was calculated.

### Rho-GTPase pull-down assay

Differentiated podocytes were serum starved (1% FBS) for 16 h prior to the experiment. Cells were stimulated with EGF (100 ng/ml) for 0, 2, or 5 min, washed once with ice-cold PBS, and lysates collected as described above. Active Rac1 and Cdc42 were pulled down using GST-CRIB beads (GE Healthcare Bio-Sciences), as described previously^16,50^. Cells were lysed with the lysis buffer (above) and mixed with the beads for 1 h at 4 °C. Beads were washed three times, and proteins were eluted from beads into SDS loading buffer.

### Wound healing assay

Undifferentiated podocytes were seeded onto a 96-well IncuCyte® ImageLock microplate coated with 0.25 μg/cm^2^ laminin 521 (CORNING) at a density of 40,000 cells per well. After cells reached a confluent state, they were serum starved in RPMI containing 1% FBS for 2 h at 37 °C. A scratch wound was made using the IncuCyte 96-well Wound Maker (Sartorius). Cells were then kept under non-permissive conditions up to 24 h and the migration rate was analyzed by IncuCyte Analysis Software (Sartorius).

### Attachment assay

Differentiated podocytes were seeded on a 96-well plate (10,000 cells per well) with laminin 521 (CORNING) and cultured under non-permissive conditions for 2 h. After 3 washes with PBS, adherent cells were fixed with 4% PFA for 15 min. Fixed cells were incubated with 0.1% crystal violet (Sigma-Aldrich) dissolved in 200 mM 3-(N-morpholino) propanesulfonic acid (BioShop) for 15 min at room temperature. After 3 washes with PBS, 10% acetic acid (Fisher Scientific) was added for 15 min. Dissolved crystal violet was quantified by the absorbance at 550 nm.

### Co-immunoprecipitation assay

Transient transfection of HEK293 cells was performed using Lipofectamine 2000 (Invitrogen) at a 1:2 DNA/Lipofectamine ratio. Cell lysates were incubated with anti-GFP antibody overnight at 4 °C, and then with protein A agarose beads (Santa Cruz, sc-2001) for 1 h at 4 °C. Beads were washed three times, and proteins were eluted from the beads into SDS loading buffer. Normal rabbit IgG (CST, 2729) was used as a negative control.

### Animals

CdGAP^*flox/flox*^ mice, which have conditional floxed exon 1 allele (C57BL/6 background), systemic CdGAP deficient mice (C57BL/6 background), and Podocin-Cre mice (mixed ICR/129/B6 background) have been described previously^27,51^. Male mice were used for experiments. Cre-negative mice were used as controls for CdGAP^*flox/flox*^;Pod-Cre (CdGAP^Pod-/-^) mice. Spot urine samples were collected at the indicated time points. To determine the genotype, mice DNA was extracted from isolated glomeruli by DNA Isolation Kit for Cells and Tissues (Roche Diagnostics, 11814770001), and subjected to PCR. The genotyping primer sequences used were as follows:

*Cre*-F 5′-gcttctgtccgtttgccg-3′.

*Cre*-R 5′-actgtgtccagaccaggc-3′.

*CdGAP* -F 5′-cctgcgctgtgcaaagagcct-3′.

*CdGAP* -R 5′-cccaaagtttaagacccgagcctc-3′.

### Plasmid constructs

Myc-tagged and GFP-tagged full-length and their truncated CdGAP constructs have been described previously^24,52^. Isoform 5 of human β-PIX was amplified by RT-PCR from mRNA from human podocytes using primers below, digested by BamHI (Cell Signaling Technology [CST], R3136) and NotI (CST, R3189), and ligated into pcDNA3.1 using T4 DNA Ligase (CST, M0202).

F 5′-accgagctcggatccatgaccgataatagcaacaatcaactggtagtaagagcaaag-3′.

R 5′-gactcgagcggccgcttatagattggtctcatcccaggcaggatcattca-3′.

Next, the constructs were digested by EcoRI (CST, R3101) and KpnI (CST, R3142) or by PspOMI (CST, R0653) and KpnI, and ligated into pEGFP-C1 and pmCherry-C1 vectors, respectively. All constructs were verified by Sanger sequencing.

### Biochemical measurements

The urinary albumin and creatinine levels were measured as previously described^16^. The former was normalized to the latter to obtain ACR. BUN and serum creatinine were measured at the Comparative Medicine and Animal Resources Centre (CMARC) of McGill University.

### In situ* hybridization*

In situ hybridization assay was performed using RNAscope (Advanced Cell Diagnostics, Inc.) with the target probe for mouse CdGAP mRNA (Advanced Cell Diagnostics, Inc., 569971) according to the manufacturer’s instructions.

### Measurement of foot process width

Transmission electron micrographs of 3 to 5 glomerular tufts per mouse were captured using an FEI Tecnai 12 BioTwin 120 kV transmission electron microscope. Analysis for foot process width has been described previously^51^.

### ADR-induced proteinuria model

CdGAP^Pod-/-^ and control mice at the age of 14–16 weeks were intravenously injected with 12 mg/kg of ADR (Sigma-Aldrich, D1515). Urine was collected at indicated time points before and after ADR treatment, and analyzed for ACR. Kidneys were harvested at 4 weeks after treatment and subjected to electron microscopic analysis.

### Statistics

All results are expressed as the mean ± standard error (SE). Statistical analyses were conducted using JMP software (SAS Institute, Cary, NC) and GraphPad Prism 9 (GraphPad Software, San Diego, CA). Multiple-group comparisons were performed using analysis of variance (ANOVA) with post-testing using the Tukey–Kramer test. Differences between two experimental values were assessed by the Student’s t-test. *P* < 0.05 was considered statistically significant.

### Study approval

The human biopsy sample was obtained from the McGill University Health Centre Kidney Disease Biorepository and the study was approved by the McGill University Health Centre Review Board. Informed consent was obtained from all study participants, and research was performed in accordance with relevant guidelines and regulations. All procedures involving mice were approved by the Animal Care Committee, McGill University and were in accordance with relevant guidelines and regulations. This study is reported in accordance with ARRIVE guidelines.

## Supplementary Information

Supplementary Information.

## Data Availability

The datasets generated during the current study are available in the PRIDE repository under the accession number PXD023718.

## Abbreviations

ACR: Albumin-to-creatinine ratio
ADR: Adriamycin
β-PIX: ARHGEF7
BUN: Blood urea nitrogen
Cdc42: Cell division control protein 42 homolog
CdGAP: Cdc42 GTPase-activating protein
ERK: Extracellular-signal regulated kinase
FA: Focal adhesion
FAK: Focal adhesion kinase
GAP: GTPase-activating protein
GDI: Guanine nucleotide dissociation inhibitor
GEF: Guanine nucleotide exchange factor
ITSN: Intersectin
pY: Phospho-tyrosine
PAS: Periodic Acid-Schiff
Rac1: Ras-related C3 botulinum toxin substrate
