# Supplementary material for: CdGAP maintains podocyte function and modulates focal adhesions in a Src kinase-dependent manner
Source: Sci Rep. 2022 Nov 4;12:18657. doi: 10.1038/s41598-022-21634-1 (PMC9636259; doi:10.1038/s41598-022-21634-1)

CdGAP maintains podocyte function and modulates focal adhesions in a Src kinase-dependent manner

Jun Matsuda, Dina Greenberg, Sajida Ibrahim, Mirela Maier, Lamine Aoudjit, Jennifer Chapelle, Cindy Baldwin, Yi He, Nathalie Lamarche-Vane, Tomoko Takano

Table of contents for supplementary information

Figure S1	Page S-2
Figure S2	Page S-3
Figure S3	Page S-4
Figure S4	Page S-5
Table S1	Page S-6
Original gels and blots	Page S-7

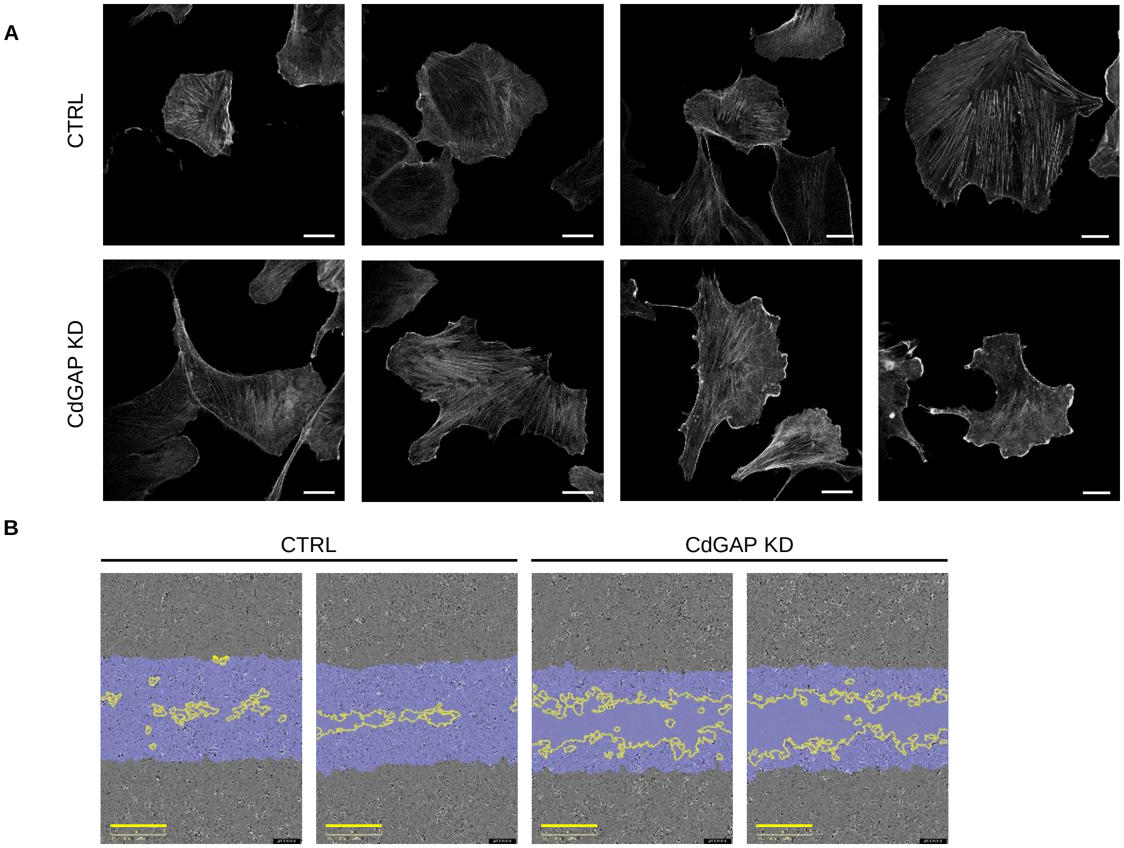

Figure S1. CdGAP depletion alters cell morphology and migration. (**A**) Immunofluorescence staining of F-actin of differentiated control (CTRL) and CdGAP KD podocytes shows altered morphology. (**B**) CdGAP KD cells demonstrate impaired migration in a scratch wound migration assay. Representative images of undifferentiated CTRL and CdGAP KD podocytes. Bar: 20 µm (**A**), 400 µm (**B**).

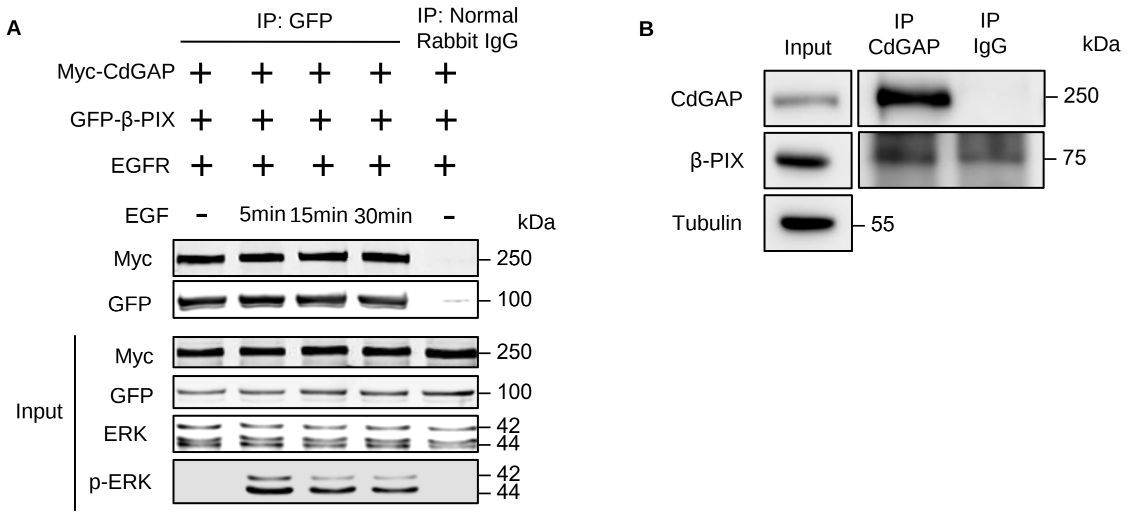

Figure S2. CdGAP and β-PIX physically interact in podocytes. (**A**) CdGAP and β-PIX interaction is not altered by EGF stimulation. HEK293 cells overexpressing myc-CdGAP and GFP-β-PIX were untreated or treated with EGF. Cell lysates were immunoprecipitated with anti-GFP antibody (for β-PIX) or normal rabbit IgG followed by immunoblotting for myc (for CdGAP). (**B**) EGF-treated podocytes were lysed and CdGAP was immunoprecipitated (IP) from cell lysates with anti-CdGAP antibodies or rabbit anti-IgG antibodies (control) followed by immunoblotting for CdGAP and β-PIX. Tubulin was used as a loading control.

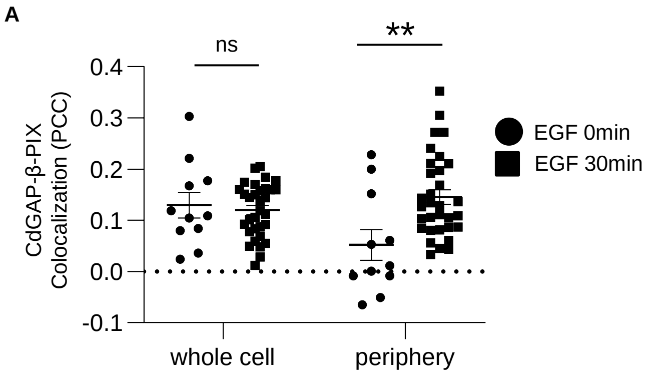

Figure S3. EGF stimulation promotes peripheral colocalization of CdGAP and β-PIX. (**A**) EGF stimulation in podocytes promotes a mild increase in peripheral colocalization of CdGAP and β-PIX. Pearson correlation coefficient (PCC) of CdGAP and β-PIX colocalization in Fig. 4A. n = 11 to 23 in each group (**A**). ns, not significant. Statistically significant differences (***P* < 0.01), assessed by the Student's t-test, are indicated.

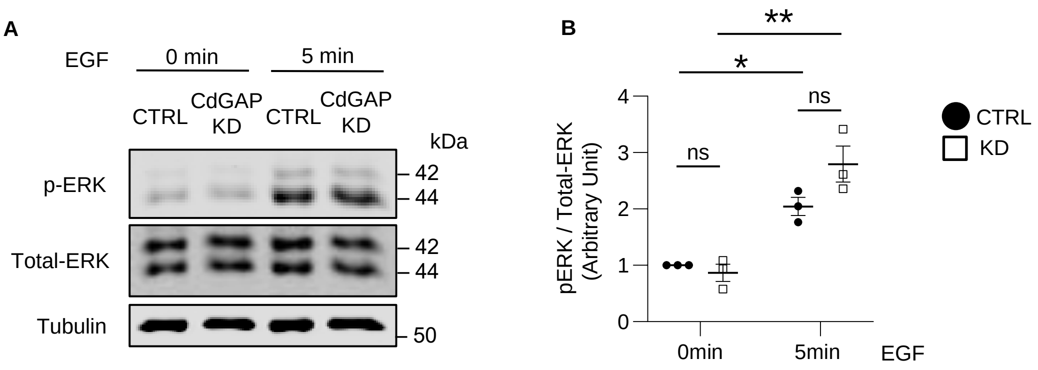

Figure S4. ERK activation in response to EGF stimulation is not affected by CdGAP depletion. Representative immunoblots for pERK and total ERK using CdGAP KD and CTRL podocytes treated with EGF (100 ng/ml). (**B**) Densitometric quantification of pERK normalized to total expression in (**A**). n = 3 (**B**) in each group. Statistically significant differences (**P* < 0.05, ***P*<0.01), assessed by ANOVA with the Tukey-Kramer test (**B**), are indicated.

Table S1. Antibodies and Reagents.

Antibodies and Reagents	Supplier and Catalog Number
Myc	Santa Cruz Biotechnology, sc-40
Streptavidin horseradish peroxidase (HRP)	abcam, ab7403
Synaptopodin	Progen, 65194
CdGAP	Sigma-Aldrich, HPA036380 for IHC, ICC and IB<br>Cell Signaling Technology (CST), 14087 for IB
Tubulin	Sigma-Aldrich, T5168
Epidermal growth factor (EGF)	BioShop, E012
Rac1	Millipore, 05-389
Cdc42	Millipore, 05-542
Extracellular signal-regulated kinase (ERK)	CST, 9102
p-ERK	CST, 9106
β-PIX	Millipore, 07-1450-I for IB and ICC<br>Santa Cruz Biotechnology, sc-393184 for ICC<br>CST, 4515 for IP
GFP	Invitrogen, A6455 for IB<br>Santa Cruz Biotechnology, sc-9996 for IB<br>Invitrogen, G10362 for IP
IgG	Invitrogen, 31887
mCherry	CST, 43590
Phosphotyrosine	Millipore, 05-321
FAK	BD Biosciences, 610088
pTyr397-FAK	CST, 8556
paxillin	Millipore, 05-417
pTyr118-paxillin	CST, 2541
Vinculin	Sigma-Aldrich, V9131
pTyr416-Src	CST, 2101
Src	Millipore, 05-184
PromoFluor-647-phalloidin	PromoKine, PK-PF647P-7-01
SU6656	Millipore, 572635

Secondary antibodies

Alexa 555-conjugated secondary anti-rabbit antibody (CST, 4413)

Alexa 488-conjugated secondary anti-mouse antibody (CST, 4408)

HRP-conjugated secondary antibodies

- Abcam, ab6789 [anti-mouse IgG], ab6721 [anti-rabbit IgG]

LiCOR secondary antibodies

- IRDye 680LT
  - 926-68050 [anti-mouse]
  - 926-68021 [anti-rabbit]
- IRDye 800CW
  - 926-32210 [anti-mouse]
  - 926-32211 [anti-rabbit]

Figure 1A

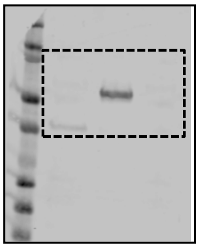

Figure 1B

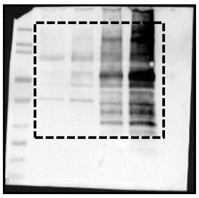

Figure 2B

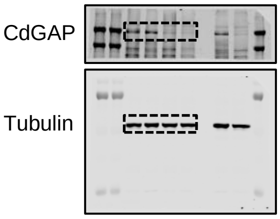

Figure 2D

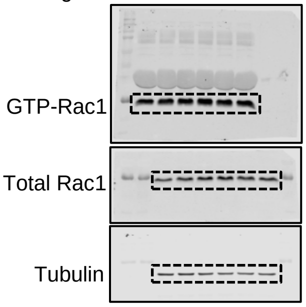

Figure 2G

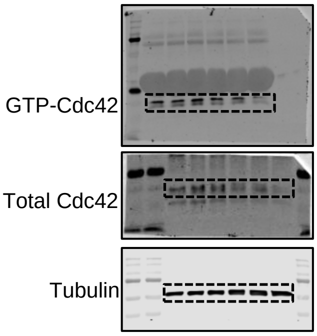

Figure 3A

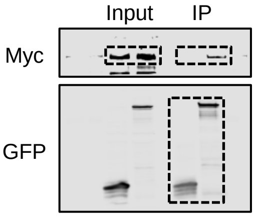

Figure 3D

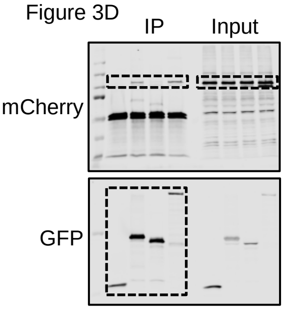

Figure 3F

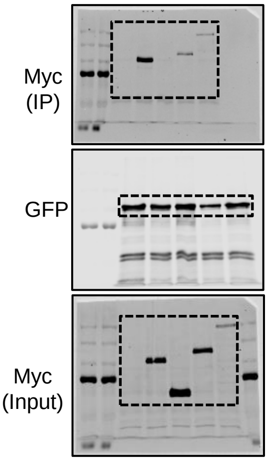

Figure 5D

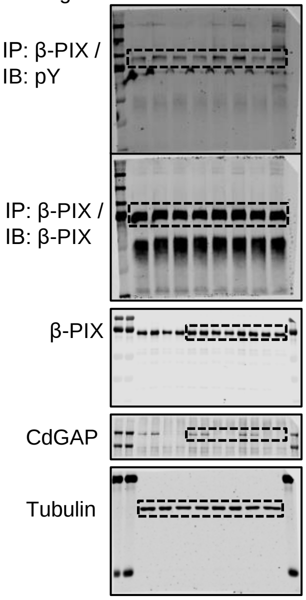

Figure 5G

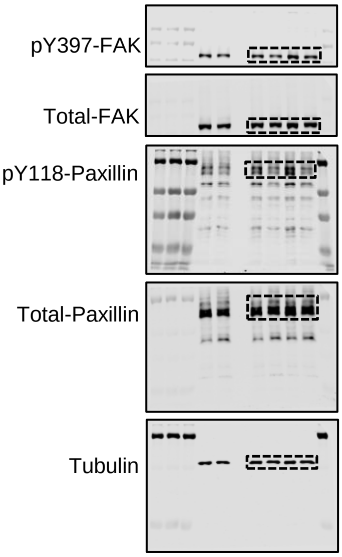

Figure 6A

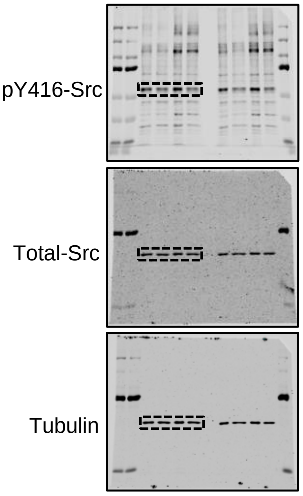

Figure 6C

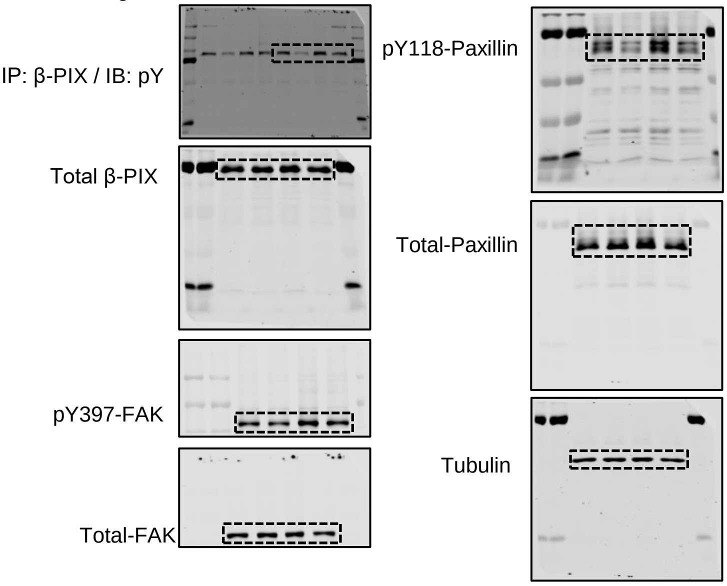

Figure 7B

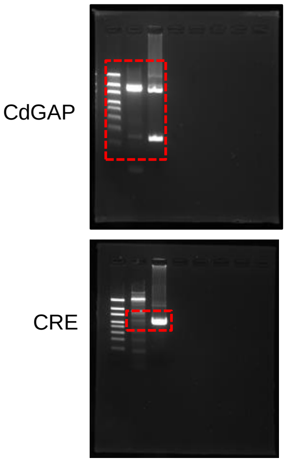

Figure S2A

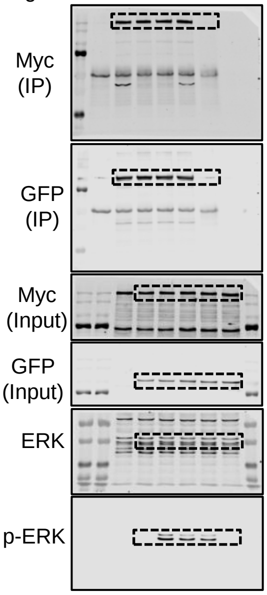

Figure S2B

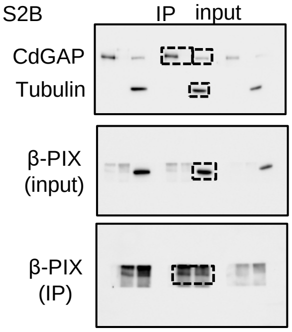

Figure S4A

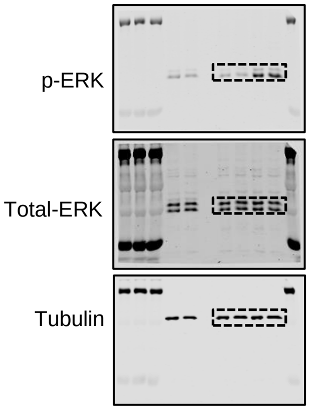

Supplement: Supplementary file 1 — Supplementary Information. [file 41598_2022_21634_MOESM1_ESM.pdf]
